# Emotional Intelligence and Physical Activity as Protective Factors Against Problematic Internet Use and Psychological Distress: A Comparative Study of Adolescents and Adults

**DOI:** 10.1192/j.eurpsy.2026.10549

**Published:** 2026-07-31

**Authors:** M. Theodoratou, G. Galani, K. Megari, K. Flora, E. Mantsos, D. Katsarou

**Affiliations:** 1 Social Sciences, Hellenic Open University, Patras; 2 Aristotle University, Thessaloniki; 3 University of Western Macedonia, Florina; 4 University of Thessaly, Volos; 5 University of Aegean, Rhodes, Greece

## Abstract

**Introduction:**

Problematic Internet Use (PIU) is an increasing mental health concern, particularly among adolescents, and is associated with anxiety, depression, and impaired functioning. Emotional intelligence (EI) may serve as a protective factor, while physical activity (PA) supports psychological well-being. This study examined the relationships among PIU, psychological distress, EI, and PA across adolescents and adults.

**Objectives:**

This study aimed to (1) investigate associations among PIU, psychological distress, EI, and PA, and (2) compare patterns of vulnerability and protective factors between adolescents and adults.

**Methods:**

A cross-sectional design was employed. Standardized self-report instruments were used: **Kessler Psychological Distress Scale (K6)** for psychological distress, **Quality of Life Scale (QOLS)** for well-being, **Internet Addiction Test (IAT) by Young** for PIU, **Rapid Assessment of Physical Activity (RAPA)** for PA, and an **Emotional Intelligence Scale** for EI. Descriptive statistics, correlation analyses, and group comparisons were conducted.

**Results:**

Among adolescents, **65% engaged in at least 30 minutes of moderate-intensity activity five times per week**, and **67% reported vigorous activity at least three times weekly**, reflecting relatively high PA levels. However, **72% displayed moderate PIU tendencies**, with frequent loss of control over online time and emotional reliance on internet use. Distress levels were elevated: over **60% reported frequent sadness, hopelessness, or insignificance**, with 38% scoring in ranges indicating risk for functional impairment. EI scores were low to moderate, particularly in recognizing others’ emotions (mean = 1.95/5).

Adults reported **53% meeting PA guidelines** but lower engagement in vigorous exercise. **58% displayed moderate PIU**, particularly difficulty disconnecting and using the internet to escape negative emotions. Psychological distress remained moderate to high, with **45% frequently reporting sadness or worry**. EI scores were slightly higher than in adolescents (mean total = 12.65/25), yet still indicated gaps in stress regulation and empathy.

Correlational analyses revealed:

- **PIU was positively associated with psychological distress** (r ≈ 0.45, p < .01).
- **EI was inversely correlated with both distress (r ≈ -0.40) and PIU (r ≈ -0.38, p < .01)**, indicating its protective role.
- **PA was negatively correlated with distress (r ≈ -0.30) and PIU (r ≈ -0.25, p < .05)**, supporting its moderating function.
- Adolescents showed stronger associations between low EI, high distress, and PIU compared to adults, highlighting developmental vulnerability.

**Conclusions:**

PIU is linked to higher distress and lower EI, particularly in adolescents. PA emerged as a protective factor. Interventions promoting EI and regular PA may reduce maladaptive internet use and improve mental health outcomes.

**Disclosure of Interest:**

None Declared

